# The Association Between Peripheral Arterial Disease and Long-Term Bleeding Events in Patients with Acute Myocardial Infarction

**DOI:** 10.3390/jcm14093183

**Published:** 2025-05-04

**Authors:** Soichiro Ban, Kenichi Sakakura, Hiroyuki Jinnouchi, Yousuke Taniguchi, Kei Yamamoto, Takunori Tsukui, Masashi Hatori, Taku Kasahara, Shun Ishibashi, Yusuke Watanabe, Masaru Seguchi, Hideo Fujita

**Affiliations:** Division of Cardiovascular Medicine, Saitama Medical Center, Jichi Medical University, 1-847 Amanuma, Omiya, Saitama City 330-8503, Japan

**Keywords:** acute myocardial infarction, peripheral artery disease, percutaneous coronary intervention, bleeding events, major adverse cardiovascular events

## Abstract

**Background**: Peripheral arterial disease (PAD) is associated with cardiovascular events in patients with acute myocardial infarction (AMI). However, there are limited reports regarding the association between PAD and bleeding events. In this study, we aimed to evaluate whether PAD is independently associated with an increased risk of major bleeding events, in addition to major adverse cardiovascular events (MACEs), in patients with AMI undergoing percutaneous coronary intervention (PCI). **Methods**: We included 1391 patients with AMI who underwent PCI and divided them into the PAD group (*n* = 210) and the non-PAD group (n = 1181). The primary endpoint was total bleeding events, defined as Bleeding Academic Research Consortium type 3/5. The secondary endpoint was MACE, defined as the composite of all-cause death, non-fatal myocardial infarction, and hospitalization for heart failure. **Results**: The median follow-up duration was 653 days. Total bleeding events were more frequently observed in the PAD group than in the non-PAD group (24.8% vs. 11.3%, *p* < 0.001). The multivariate Cox hazard analysis confirmed that PAD was significantly associated with total bleeding events (HR 1.509; 95% CI 1.056–2.156, *p* = 0.024) as well as MACEs (HR 2.152; 95% CI 1.510–3.066, *p* < 0.001) after controlling for confounding factors. **Conclusions**: PAD was independently associated with a higher risk of major bleeding and cardiovascular events in patients with AMI undergoing PCI. These findings suggest that PAD should be recognized as a critical factor in risk stratification for AMI and may affect individualized bleeding risk management strategies in patients with AMI.

## 1. Introduction

Acute myocardial infarction (AMI) remains a critical health issue globally and is characterized by significant morbidity and mortality [1,2]. The development of primary percutaneous coronary intervention (PCI) has revolutionized the treatment of AMI, resulting in better clinical outcomes [3]. However, the success of primary PCI is sometimes tempered by the occurrence of complications such as bleeding, which can significantly impact all-cause mortality [4,5].

Bleeding events after PCI are common and associated with increased mortality and poor clinical outcomes [6,7]. The incidence of bleeding events after PCI is influenced by the presence of high-bleeding risk factors. Recently, the Japanese Society of Cardiology published the Japanese version of the High Bleeding Risk criteria (J-HBR) [8]. One of the major criteria in the J-HBR is peripheral artery disease (PAD), which was not focused on in other risk criteria, such as the Academic Research Consortium for High Bleeding Risk (ARC-HBR) [9]. Although recent international studies, including the EPICOR Asia study, have investigated the long-term bleeding risk in patients with acute coronary syndrome, the association between PAD and bleeding has not been established in patients with AMI [10]. The J-MINUET registry reported that PAD was associated with worse clinical outcomes in patients with AMI [11]. Most of these studies primarily focused on ischemic outcomes, and few have comprehensively evaluated the association between PAD and bleeding in patients with AMI. The purpose of this study was to investigate the association between PAD and major bleeding in patients with AMI after primary PCI.

## 2. Materials and Methods

### 2.1. Study Design

We reviewed all patients with AMI treated at our institution (Saitama Medical Center, Jichi Medical University) from January 2015 to December 2022. The inclusion criterion was patients with AMI. The exclusion criteria were as follows: (1) patients without complete ankle–brachial index (ABI) measurement during hospitalization, (2) a second or more than two AMIs during the study period, (3) patients who underwent CABG during hospitalization, and (4) patients who did not undergo PCI to the culprit lesion of AMI. These criteria aimed to ensure a uniform study population for evaluating the relationship between PAD and bleeding. Patients without ABI data were excluded for diagnostic consistency, while those with CABG or those without PCI were excluded to reduce potential confounding factors.

We defined PAD as a history of surgery or EVT for PAD, ABI < 0.9, or inter-arm blood pressure difference (IABPD) ≥ 10 mmHg [12,13,14,15]. Patients with only symptoms suggestive of PAD were not classified as having PAD. The final study population was divided into a PAD group and a non-PAD group. The primary endpoint was total bleeding events, which is defined as type 3 or 5 bleeding events by the Bleeding Academic Research Consortium (BARC) [9]. BARC type 1, 2, and 4 bleeding were not included in total bleeding events. The secondary endpoint was major cardiovascular events (MACEs), which was defined as the composite of all-cause death, non-fatal myocardial infarction, and readmission for heart failure. Hospital records were used to obtain information regarding the clinical outcomes. The day of PCI was defined as the index day (day 1). The study patients were followed up until all-cause death or until the study end date (31 May 2023). This study was approved by the institutional review board of the Saitama Medical Center, Jichi Medical University (S22-074), and the need for written informed consent was waived because of the retrospective study design.

### 2.2. Definitions

AMI was defined according to the universal definition [16,17]. Diagnostic ST elevation was defined as new ST elevation at the J point in at least two contiguous leads of 2 mm (0.2 mV), and patients with ST elevation were diagnosed as having STEMI [18,19]. Definitions of hypertension, diabetes mellitus, and dyslipidemia are described elsewhere [20,21,22]. We used the laboratory data at admission [21]. Left ventricular ejection fraction (LVEF) was measured via transthoracic echocardiography during the index hospitalization [23]. We also calculated estimated glomerular filtration rate (eGFR) [24]. The initial and final thrombolysis in myocardial infarction (TIMI) flow grades were documented from invasive coronary angiography [25].

### 2.3. Statistical Analysis

Data are expressed as the median (Q1–Q3) or percentage. Categorical variables are presented as numbers (percentages) and were compared using Fisher’s exact test. The Shapiro–Wilk test was performed to examine whether the continuous variables were normally distributed or not. Because none of the variables were normally distributed, continuous variables were compared using the Mann–Whitney U test. Event-free survival curves were constructed using the Kaplan–Meier method, and statistical differences were examined with the log-rank test. We performed a multivariate Cox hazard analysis to investigate the association between PAD and total bleeding events or between PAD and MACEs after controlling for confounding factors. In the model, total bleeding events or MACEs were used as the dependent variable. Variables that were significantly different (*p* < 0.05) between the PAD and non-PAD groups were included as independent variables in the model. Variables with missing values were not included in the model. To avoid multicollinearity, similar variables were not entered simultaneously. Hazard ratios (HRs) and the 95% confidence intervals (CIs) were calculated. A *p*-value < 0.05 was considered statistically significant. Furthermore, we conducted propensity score matching as a supplemental analysis. A logistic regression analysis was performed to calculate the propensity score using the full database. In this model, PAD was set as a dependent variable, whereas age, sex, BMI, presence of diabetes mellitus, chronic renal failure on hemodialysis, history of PCI, history of CABG, history of stroke, hemoglobin level, STEMI, and LVEF were set as independent variables. For matching, the match tolerance was set as a width of 0.25 multiplied by the SD of the propensity score distribution. Case–control matching resulted in 202 fuzzy matches with maximized matching performance. Thus, these 202 pairs were used in the supplementary analysis. All analyses were performed using statistical software, SPSS 25/Windows (SPSS, Chicago, IL, USA).

## 3. Results

**Figure 1:**
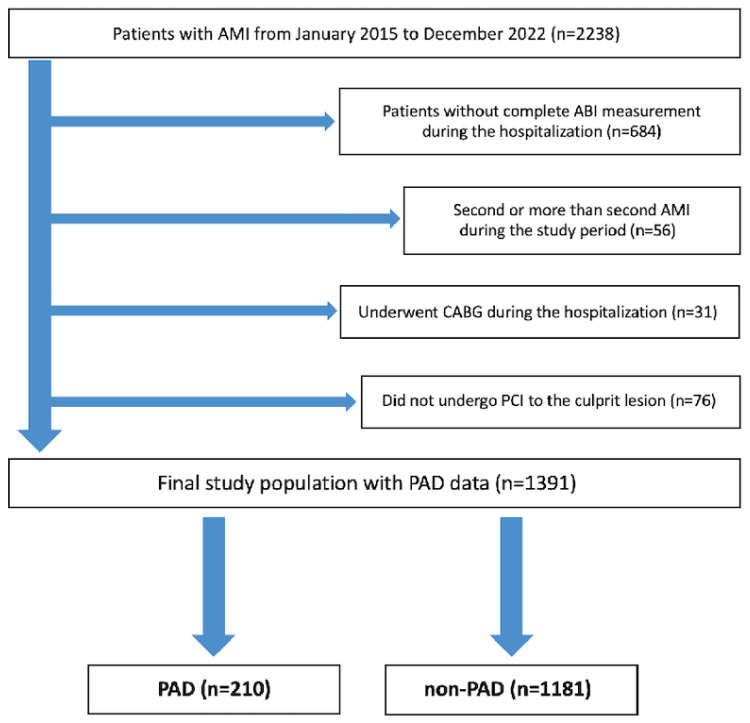
Study flowchart. Abbreviations: AMI = acute myocardial infarction, ABI = ankle–brachial index, CABG = coronary artery bypass grafting, PCI = percutaneous coronary intervention, and PAD = peripheral arterial disease.

From January 2015 to December 2022, 2238 patients with AMI were admitted to our institution. After excluding 847 patients who met the exclusion criteria, the final study population consisted of 1391 patients with AMI, who were assigned to the PAD group (n = 210) or the non-PAD group (n = 1181) (Figure 1).

The comparison of patients’ characteristics between the two groups is shown in Table 1. Age was older and body mass index was lower in the PAD group compared to the non-PAD group. Anemia, hemodialysis, and history of cerebral infarction were more frequently observed in the PAD group than in the non-PAD group. The prevalence of STEMI was significantly lower in the PAD group than in the non-PAD group. Aspirin, thienopyridine, statin, and antihypertensive medications at admission were more frequently prescribed in the PAD group. Oral antidiabetics and insulin were also more prescribed in the PAD group. Table 2 shows the comparison of angiographic and procedural findings between the 2 groups. Triple vessel disease, left main disease, first TIMI flow grade 3, and chronic total occlusion in non-culprit arteries were more frequently observed in the PAD group than in the non-PAD group. Left main disease was also more frequently found in the PAD group.

**Figure 2:**
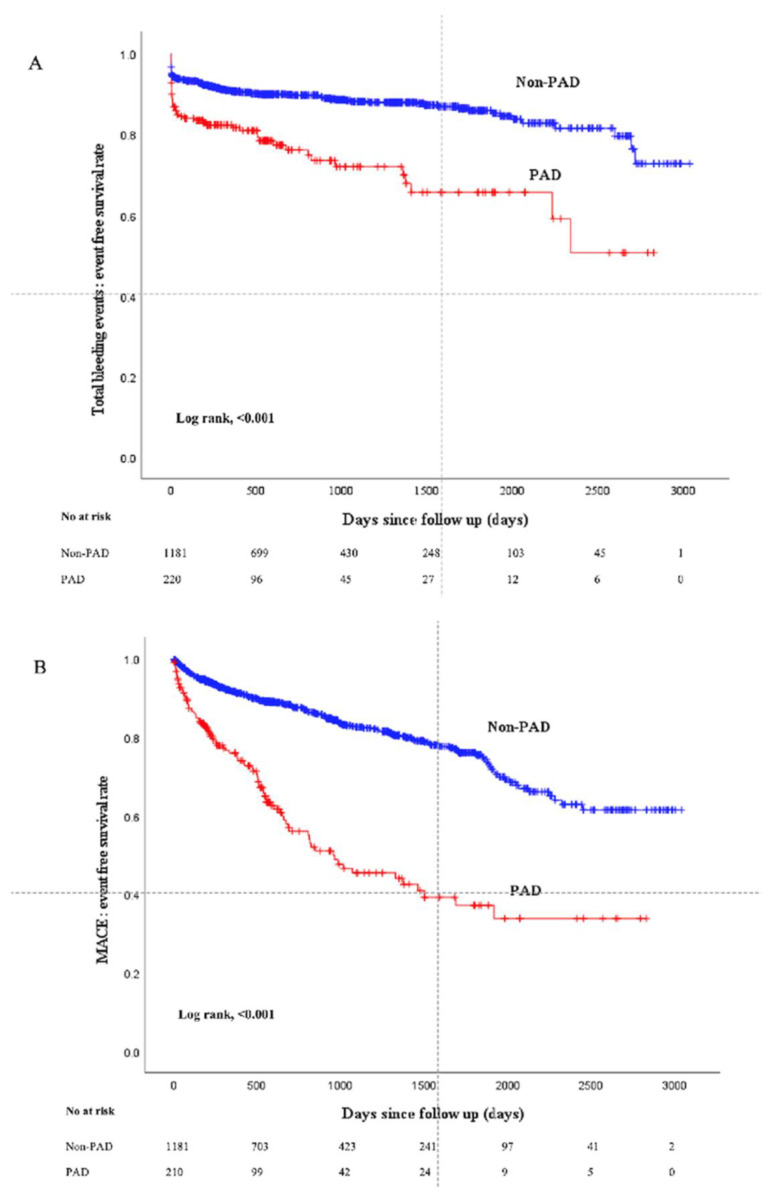
Kaplan–Meier curves for total bleeding or MACE-free survival events-free survival between the PAD group and the non-PAD group. (**A**) Comparison of total bleeding events. (**B**) Comparison of MACEs. Abbreviations: MACEs = major cardiovascular events.

Figure 2 shows the Kaplan–Meier curves between the two groups. The median follow-up duration was 653 (Q1: 259–Q3: 1404) days. Total bleeding events were more frequently observed in the PAD group compared to the non-PAD group (log-rank *p* < 0.001), and MACEs were also more frequently observed in the PAD group (log-rank *p* < 0.001). Table 3 shows the comparison of clinical outcomes between the two groups. A total of 186 bleeding events were observed during the follow-up duration. The incidence of total bleeding events was significantly higher in the PAD group than in the non-PAD group. In particular, the incidence of BARC type 3 bleeding events was significantly higher in the PAD group than in the non-PAD group, whereas there was no significant difference in BARC type 5 bleeding events between the 2 groups. The incidence of PCI access site-related bleeding events was significantly higher in the PAD group than in the non-PAD group. The incidence of MACEs was significantly higher in the PAD group than in the non-PAD group. The results of multivariate Cox hazard analysis are shown in Table 4. PAD was significantly associated with total bleeding events (HR 1.509, 95% CI 1.056–2.156, *p* = 0.024) and MACEs (HR 2.152, 95% CI 1.510–3.066, *p* < 0.001) after controlling for multiple confounding factors including age, gender, overweight (BMI ≥ 25), anemia, chronic renal failure on hemodialysis, previous myocardial infarction, previous cerebral infarction, CRP levels, STEMI, Killip class, diastolic blood pressure at admission, LVEF, use of NPPV, number of narrowed coronary arteries, first TIMI flow grade, use of drug-eluting stents, and catheter size.

Supplemental Tables S1–S3 show the comparison of clinical, lesion, and procedural outcomes between the matched PAD and matched non-PAD groups, respectively. Supplementary Figure S1 shows the Kaplan–Meier curves for bleeding (A) and MACEs (B) after propensity score matching. Total bleeding events were more frequently observed in the matched PAD group than in the matched non-PAD group without reaching statistical significance, whereas MACEs were more frequently observed in the matched PAD group.

## 4. Discussion

We included 1391 patients with AMI and divided them into the PAD group (n = 210) and the non-PAD group (n = 1181). We followed up the patients with a median duration of 653 days. Total bleeding events were more frequently observed in the PAD group than in the non-PAD group. The multivariate Cox hazard analysis revealed that PAD was significantly associated with total bleeding events (HR 1.509, 95% CI 1.056–2.156, *p* = 0.024) and MACEs (HR 2.152, 95% CI 1.510–3.066, *p* < 0.001) after controlling for multiple confounding factors. In the propensity score matching analysis, total bleeding events were more frequently observed in the matched PAD group than in the matched non-PAD group without reaching statistical significance, whereas MACEs were more frequently observed in the matched PAD group.

We should clarify the difference between the present study and previous studies. Saw et al. conducted a pooled analysis of eight randomized PCI trials and revealed a trend toward higher major bleeding in patients with PAD (4.5%) as compared to those without PAD (3.9%) (*p* = 0.06) [26]. Gupta et al. showed that a history of PAD was associated with ischemic and bleeding outcomes 2 years after successful PCI (HR 1.60%, 95% CI: 1.31–1.96; *p* < 0.0001) [27]. Bashar et al. also investigated the association between extracardiac vascular disease (ECVD), including PAD, and clinical outcomes after PCI and found that ECVD was associated with worse outcomes in patients undergoing PCI, including significantly higher rates of death and stroke [28]. Gao et al. investigated the impact of PAD on MACEs and bleeding in patients undergoing complex or non-complex PCI and found that PAD was associated with increased risk of bleeding regardless of procedural complexity [29]. Pinxterhuis et al. conducted a three-year pooled patient-level data analysis of two randomized PCI trials, including 5989 all-comer patients, and revealed that PCI patients with PAD had a significantly higher bleeding risk than PCI patients without PAD [30]. These studies did not focus on patients with AMI, whereas we focused on patients with AMI. Because patients with AMI have a greater risk of bleeding than patients with chronic coronary syndrome [31], it is important to elucidate the association between PAD and bleeding in patients with AMI who have undergone PCI.

In our study, PAD was significantly associated with MACEs, including all-cause death, non-fatal myocardial infarction, and readmission for heart failure. PAD is characterized by systemic atherosclerosis and extensive vascular damage, including the coronary arteries [32,33]. In patients with AMI, the presence of PAD, irrespective of symptoms, is strongly associated with an increased risk of MACEs, including all-cause death, myocardial infarction, and readmission for heart failure [12,34]. Patients with advanced PAD are more likely to be frail and undernourished [35]. Reduced physical activity and increased frailty might be associated with increased risk of MACEs [36].

We should discuss why PAD is associated with long-term bleeding in patients with AMI. Patients with PAD undergoing PCI for AMI are associated with long-term bleeding risks due to several interrelated factors. Among patients undergoing coronary stenting, those with PAD have more ischemic events, including revascularization, than those without PAD. Thus, patients with PAD are more likely to undergo multiple PCI procedures and require longer dual antiplatelet therapy. As a result, patients with PAD tend to have more bleeding events [37]. The systemic inflammation and endothelial dysfunction that are prevalent in PAD may further exacerbate bleeding risk, especially under antithrombotic treatment [38]. Moreover, patients with PAD often have comorbidities such as diabetes mellitus and hypertension, which contribute to an increased risk of bleeding [39]. Although PAD is a common disease worldwide, the optimal type, dose, and timing of antiplatelet and anticoagulant medications have not been determined. There are no uniform guidelines on this topic. In patients with PAD after revascularization, DAPT or the combination of aspirin and low-dose rivaroxaban may reduce the incidence of ischemic events but increase bleeding [40,41]. Therefore, patients with a high risk of bleeding should have an appropriate risk index.

The clinical implications of the present study should be noted. Since PAD in patients with AMI is associated with long-term bleeding events, it is important to recognize PAD as a risk factor for bleeding through routine measurement of ABI. It may be reasonable to include ABI in the standard practice for patients with AMI, because ABI is a non-invasive and non-expensive test. These high-risk patients should be carefully followed up by cardiologists. Careful follow-up may include close monitoring of hemoglobin levels and gastrointestinal symptoms. We should consider switching from DAPT to short DAPT in patients with PAD due to the increased risk of bleeding. Given the higher bleeding risk and lower thrombotic risk in Japanese and other East Asian populations compared to Western populations [42,43], the strategy to minimize bleeding risk is essential to improve the overall outcomes of AMI patients with PAD. Although the Japanese version of the HBR (J-HBR) includes PAD as one of the major criteria for bleeding [8], major bleeding risk criteria or bleeding risk score, including ARC-HBR and PRECISE DAPT, do not deem PAD as a significant risk factor [9,44]. Future bleeding risk criteria or revisions of major bleeding risk criteria, which would influence antiplatelet therapy decisions, may consider including PAD as one of the criteria.

There are several limitations to the present study. Since this study is a single-center, retrospective study, there is a potential for selection bias. To address this, we performed multivariable Cox proportional hazards regression analysis to adjust for known confounding variables. Furthermore, we also performed propensity score matching to adjust for potential confounding factors. Although the propensity score matching adjusted for clinical background, this matching reduced the study population significantly from 1391 to 404, which created a risk of beta-error. In the propensity score matched cohort, total bleeding events were not statistically significant between the matched PAD and non-PAD groups, which might be affected by beta error. Long-term bleeding events might have been influenced by post-discharge medications. Since our institution is a tertiary university hospital, most patients were referred to their local clinics after discharge. Because patients received their medications, including antiplatelet therapy, from these clinics, we were unable to obtain detailed information on post-discharge medications, including DAPT. Although the recommended DAPT duration was described in referral letters according to the guidelines, adherence to these recommendations was not systematically monitored. It is possible that some patients continued DAPT beyond the recommended period, which might have led to an increased incidence of bleeding events. This lack of information regarding post-discharge medications is a major limitation of this study. Frailty might be more prevalent in patients with PAD and could have affected clinical outcomes because frailty is closely associated with bleeding events [45]. However, since specific metrics to assess frailty were not available in our dataset, we could not evaluate or adjust for frailty in our multivariate analysis, which represents another important limitation. Because ABI and IABPD were measured in the physiological laboratory, the most severe patients, such as those with cardiogenic shock requiring mechanical support who could not be transported to the physiological laboratory, did not have their ABI and IABPD measured [46]. As a result, the most severely ill patients might not be included in our analysis, potentially limiting the generalizability of our findings. Furthermore, no imputation methods were used for missing data in this study.

## 5. Conclusions

In this study, PAD was significantly associated with major bleeding as well as adverse cardiovascular events in patients with AMI who underwent PCI. The presence of PAD needs to be recognized as a risk factor for bleeding in patients with AMI. The authors of future studies should validate our findings in broader cohorts and assess whether recognizing PAD as a bleeding risk can improve clinical outcomes in patients with AMI.

## Figures and Tables

**Table 1:** Comparison of clinical characteristics between the PAD group and the non-PAD group.

	All (n = 1391)	PAD (n = 210)	Non-PAD (n = 1181)	*p*-Value
Age, years	71.0 (61.0–78.0)	77.0 (70.0–82.3)	70.0 (60.0–78.0)	<0.001
Male, n (%)	1099 (79.0)	156 (74.3)	943 (79.8)	0.080
Body mass index (kg/m^2^)	23.8 (21.6–26.0)	22.8 (20.6–25.4)	23.9 (21.8–26.1)	<0.001
Ankle–brachial index	1.11 (1.01–1.18)	0.78 (0.65–0.90)	1.13 (1.05–1.19)	<0.001
Ankle–brachial pulse wave velocity (cm/s)	1606 (1379–1923) (n = 1383)	1796 (1459–2293) (n = 206)	1588 (1361–1876) (n = 1177)	<0.001
Inter-arm blood pressure difference (mmHg)	2.0 (1.0–4.0) (n = 1361)	4.0 (2.0–10.0) (n = 197)	2.0 (1.0–4.0) (n = 1164)	<0.001
Current smoker, n (%)	474 (34.2) (n = 1386)	68 (32.5) (n = 209)	406 (34.5) (n = 1177)	0.635
Comorbidities				
Hypertension, n (%)	1127 (81.0)	180 (85.7)	947 (80.2)	0.069
Hyperlipidemia, n (%)	831 (59.7)	134 (63.8)	697 (59.0)	0.195
Diabetes mellitus, n (%)	592 (42.6)	109 (51.9)	483 (40.9)	0.003
Anemia, n (%)	413 (29.7)	104 (49.5)	309 (26.2)	<0.001
Atrial fibrillation, n (%)	198 (14.2)	37 (17.6)	161 (13.6)	0.134
Chronic renal failure on hemodialysis, n (%)	47 (3.4)	19 (9.0)	28 (2.4)	<0.001
History of previous PCI, n (%)	247 (17.8)	61 (29.0)	186 (15.7)	<0.001
History of previous CABG, n (%)	39 (2.8)	11 (5.2)	28 (2.4)	0.037
History of previous EVT, n (%)	43 (3.3)	43 (20.5)	0 (0.0)	<0.001
History of previous MI, n (%)	167 (12.0)	40 (19.0)	127 (10.8)	0.001
History of cerebral infarction, n (%)	135 (9.7)	35 (16.7)	100 (8.5)	<0.001
History of PAD surgery, n (%)	23 (1.7)	23 (11.0)	0 (0.0)	<0.001
Laboratory data				
Serum creatinine (mg/dL)	0.85 (0.69–1.05)	1.04 (0.80–1.52)	0.83 (0.68–1.000)	<0.001
eGFR, (mL/min/1.73 m2)	65.9 (49.8–81.0)	49.6 (34.0–66.8)	68.5 (53.7–82.7)	<0.001
Hemoglobin levels (g/dL)	13.7 (12.4–15.0)	12.7 (11.1–14.0)	13.9 (12.7–15.2)	<0.001
Brain natriuretic peptide (pg/mL)	106.0 (35.5–397.3) (n = 1378)	498.4 (108.1–1029.5) (n = 206)	90/2 (32.3–287.2) (n = 1172)	<0.001
Peak creatine kinase (U/L)	702.0 (207.8–2059.0)	393.5 (148.5–1269.0)	778.0 (225.8–2137.5)	<0.001
Peak creatine kinase-MB (U/L)	56.0 (11.0–205.0) (n = 1389)	25.0 (8.0–124.0)	62.0 (13.0–215.0) (n = 1179)	<0.001
Hemoglobin A1c (%)	6.1 (5.7–7.0) (n = 1384)	6.3 (5.8–7.2) (n = 206)	6.1 (5.7–7.0) (n = 1178)	0.015
Platelets, (×103/μL)	21.9 (18.2–26.6)	22.0 (17.9–28.2)	21.9 (18.2–26.6)	0.775
C-reactive protein (mg/μL)	0.20 (0.09–0.79)	0.51 (0.17–2.46)	0.18 (0.09–0.59)	<0.001
Type of acute myocardial infarction				
STEMI, n (%)	811 (58.3)	82 (39.0)	729 (61.7)	<0.001
NSTEMI, n (%)	580 (41.7)	128 (61.0)	452 (38.3)	
Cardiopulmonary arrest out of hospital, n (%)	47 (3.4)	5 (2.4)	42 (3.6)	0.533
Killip classification of 1 or 2, n (%)	1145 (82.3)	149 (71.0)	996 (84.3)	<0.001
Killip classification of 3 or 4, n (%)	246 (17.7)	61 (29.0)	185 (15.7)	
Cardiogenic shock at admission, n (%)	103 (7.4)	22 (10.5)	81 (6.9)	0.084
Vital sings				
Systolic blood pressure at admission (mmHg)	141.0 (122.0–163.0)	140.0 (117.8–163.0)	142.0 (123.0–163.0)	0.279
Diastolic blood pressure at admission (mmHg)	83.0 (71.0–97.0)	79.0 (66.0–92.0)	84.0 (72.0–98.0)	<0.001
Heart rate at admission (bpm)	80.0 (67.0–96.0)	85.0 (67.8–102.3)	79.0 (67.0–95.0)	0.003
Left ventricular ejection fraction (%)	54.0 (42.0–62.7)	47.1 (35.6–61.7)	55.0 (43.5–63.0)	<0.001
Medication at admission				
Aspirin, n (%)	350 (25.8) (n = 1356)	86 (41.5) (n = 207)	264 (23.0) (n = 1149)	<0.001
Thienopyridine, n (%)	197 (14.5) (n = 1356)	63 (30.4) (n = 207)	134 (11.7) (n = 1149)	<0.001
Statins, n (%)	445 (32.8) (n = 1356)	98 (47.3) (n = 207)	347 (30.2) (n = 1149)	<0.001
ACE inhibitors or ARBs, n (%)	523 (38.6) (n = 1355)	112 (54.1) (n = 207)	411 (35.8) (n = 1148)	<0.001
Beta-blockers, n (%)	306 (22.6) (n = 1355)	74 (35.7) (n = 207)	232 (20.2) (n = 1148)	<0.001
Calcium channel blocker, n (%)	501 (37.0) (n = 1355)	97 (46.9) (n = 207)	404 (35.2) (n = 1148)	0.002
Diuretics, n (%)	232 (17.1) (n = 1355)	60 (29.0) (n = 207)	172 (15.0) (n = 1148)	<0.001
Oral antidiabetic, n (%)	370 (27.3) (n = 1355)	75 (36.2) (n = 207)	295 (25.7) (n = 1148)	0.001
Insulin, n (%)	78 (5.8) (n = 1355)	20 (9.7) (n = 207)	58 (5.1) (n = 1148)	0.014
Direct oral anticoagulants, n (%)	44 (3.2) (n = 1355)	10 (4.8) (n = 207)	34 (3.0) (n = 1148)	0.197
Warfarin, n (%)	29 (2.1) (n = 1355)	5 (2.4) (n = 207)	24 (2.1) (n = 1148)	0.793
Mechanical complications after PCI				
Ventricular septal perforation, n (%)	0 (0.0)	0 (0.0)	0 (0.0)	-
Cardiac free wall rupture, n (%)	3 (0.2)	0 (0.0)	3 (0.3)	1.000
Papillary muscle rupture, n (%)	4 (0.3)	1 (0.5)	3 (0.3)	0.481
Mechanical circulatory support				
PCPS, n (%)	21 (1.5)	2 (1.0)	19 (1.6)	0.758
Intra-aortic balloon pumping, n (%)	408 (29.3)	68 (32.4)	340 (28.8)	0.324
Impella, n (%)	5 (0.4)	0 (0.0)	5 (0.4)	1.000
Medical therapy during hospitalization				
Temporary pacing, n (%)	54 (3.9)	10 (4.8)	44 (3.7)	0.441
Mechanical ventilation, n (%)	88 (6.3)	18 (8.6)	70 (5.9)	0.165
NPPV, n (%)	112 (8.1)	32 (15.2)	80 (6.8)	<0.001
Continuous hemofiltration, n (%)	18 (1.3)	6 (2.9)	12 (1.0)	0.042

Data are expressed as the median (Q1–Q3) or numbers (percentages). The Mann–Whitney U test was used for abnormally distributed continuous variables. Fisher’s exact probability test was used for categorical variables. Abbreviations: PAD = peripheral arterial disease, PCI = percutaneous coronary intervention, CABG = coronary artery bypass grafting, EVT = endovascular therapy, MI = myocardial infarction, eGFR = estimated glomerular filtration rate, STEMI = ST-segment elevation myocardial infarction, NSTEMI = non-ST-segment elevation myocardial infarction, ACE inhibitors = angiotensin-converting enzyme inhibitors, ARB = angiotensin receptor blockers, PCPS = percutaneous cardiopulmonary support, and NPPV = non-invasive positive pressure ventilation.

**Table 2:** Comparison of lesion and procedural characteristics between the PAD group and the non-PAD group.

	All (n = 1391)	PAD (n = 210)	Non-PAD (n = 1181)	*p*-Value
Number of narrowed coronary arteries				0.002
Single, n (%)	664 (46.3)	87 (36.7)	567 (48.0)	
Double, n (%)	449 (32.3)	71 (33.8)	378 (32.0)	
Triple, n (%)	298 (21.4)	62 (29.5)	236 (20.0)	
Infarct-related artery				0.727
Left main–left anterior descending artery, n (%)	706 (50.8)	102 (48.6)	604 (51.1)	
Right coronary artery, n (%)	471 (33.9)	77 (36.7)	394 (33.4)	
Left circumflex artery, n (%)	205 (14.7)	30 (13.6)	175 (14.9)	
Graft, n (%)	9 (0.6)	2 (0.9)	7 (0.6)	
50% ≥ stenosis at the left main coronary trunk, n (%)	132 (9.5)	33 (15.7)	99 (8.4)	0.002
First TIMI flow (0,1,2,3)				<0.0001
0, n (%)	523 (37.6)	56 (26.7)	467 (39.5)	
1, n (%)	94 (6.8)	9 (4.3)	85 (7.2)	
2, n (%)	250 (18.0)	38 (18.1)	212 (18.0)	
3, n (%)	524 (37.7)	107 (51.0)	417 (35.3)	
Final TIMI flow (0,1,2,3)				0.754
0, n (%)	7 (0.5)	1 (0.5)	6 (0.5)	
1, n (%)	9 (0.6)	2 (1.0)	7 (0.6)	
2, n (%)	48 (3.5)	5 (2.4)	43 (3.6)	
3, n (%)	1327 (95.4)	202 (96.2)	1125 (95.3)	
Chronic total occlusion in non-culprit arteries, n (%)	190 (13.7)	46 (21.9)	144 (12.2)	<0.001
Use of aspiration catheter, n (%)	168 (12.1)	14 (6.7)	154 (13.0)	0.008
Final PCI procedure				<0.001
Plain old balloon angioplasty, n (%)	43 (3.1)	7 (3.3)	36 (3.0)	
Drug-coated balloon, n (%)	85 (6.1)	28 (13.3)	57 (4.8)	
Bare metal stent, n (%)	14 (1.0)	3 (1.4)	11 (0.9)	
Drug-eluting stent, n (%)	1228 (88.3)	169 (80.5)	1059 (89.7)	
POBA and thrombectomy, n (%)	9 (0.6)	0 (0.0)	9 (0.8)	
Aspiration only, n (%)	7 (0.5)	2 (1.0)	5 (0.4)	
Wire did not cross the lesion, n (%)	5 (0.4)	1 (0.5)	4 (0.3)	
Approach site				<0.001
Radial, n (%)	1032 (74.2)	127 (60.5)	905 (76.6)	
Brachial, n (%)	14 (1.0)	9 (4.3)	5 (0.4)	
Femoral, n (%)	345 (24.8)	74 (35.2)	261 (22.9)	
Catheter size (Fr)				<0.001
6 Fr, n (%)	1010 (72.6)	125 (59.5)	885 (74.9)	
7 Fr, n (%)	367 (26.4)	81 (38.6)	286 (24.2)	
8 Fr, n (%)	14 (1.0)	4 (1.9)	10 (0.8)	

Data are expressed as the median (Q1–Q3) or numbers (percentages). Fisher’s exact probability test was used for categorical variables. Abbreviations: TIMI = thrombolysis in myocardial infarction.

**Table 3:** The comparison of clinical outcomes between the PAD group and the non-PAD group.

	All (n = 1391)	PAD (n = 210)	Non-PAD (n = 1181)	*p*-Value
Total bleeding event, n (%)	186 (13.4)	52 (24.8)	134 (11.3)	<0.001
BARC type 3 bleeding, n (%)	151 (10.9)	42 (20.0)	109 (9.2)	<0.001
- BARC type 3a bleeding, n (%)	108 (7.8)	29 (13.8)	79 (6.7)	0.001
- BARC type 3b bleeding, n (%)	64 (4.6)	19 (9.0)	45 (3.8)	0.002
- BARC type 3c bleeding, n (%)	9 (0.6)	0 (0.0)	9 (0.8)	0.371
BARC type 5 bleeding, n (%)	38 (2.7)	6 (2.9)	32 (2.7)	0.820
- BARC type 5a bleeding, n (%)	15 (1.1)	3 (1.4)	12 (1.0)	0.485
- BARC type 5b bleeding, n (%)	23 (1.7)	3 (1.4)	20 (1.7)	1.0000
Bleeding site				
Gastrointestinal bleeding, n (%)	44 (3.2)	9 (4.3)	35 (3.0)	0.289
Intra-abdominal bleeding, n (%)	12 (0.9)	3 (1.4)	9 (0.8)	0.406
Access site-related bleeding, n (%)	27 (1.9)	12 (5.7)	15 (1.3)	<0.001
Intracranial bleeding, n (%)	5 (0.4)	1 (0.5)	4 (0.3)	0.559
Required VA-ECMO, n (%)	20 (1.4)	3 (1.4)	17 (1.4)	1.000
Hematuria, n (%)	9 (0.6)	4 (1.9)	5 (0.4)	0.034
Others, n (%)	64 (4.6)	19 (9.0)	45 (3.8)	0.002
MACEs, n (%)	278 (20.0)	86 (41.0)	192 (16.3)	<0.001
All-cause death, n (%)	117 (8.4)	39 (18.6)	78 (6.6)	<0.001
- Cardiac death, n (%)	46 (3.3)	22 (10.5)	24 (2.0)	<0.001
Non-fatal myocardial infarction, n (%)	97 (7.0)	26 (12.4)	71 (6.0)	0.002
Readmission for heart failure, n (%)	135 (9.7)	47 (22.4)	88 (7.5)	<0.001

Data are expressed as the median (Q1–Q3) or numbers (percentages). Fisher’s exact probability test was used for categorical variables. Abbreviations: BARC = Bleeding Academic Research Consortium, VA-ECMO = veno-arterial extracorporeal circulatory membrane oxygenation, and MACEs = major adverse cardiovascular events.

**Table 4:** (**a**) Multivariate Cox hazard model to predict total bleeding events. (**b**) Multivariate Cox hazard model to predict MACEs.

**(a)**			
**Composite endpoint**	**Hazard ratios**	**95% confidence interval**	***p*-value**
Total bleeding events			
PAD group	Reference		
Unadjusted PAD group	2.515	1.824–3.468	<0.001
Adjusted PAD group	1.509	1.056–2.156	0.024
**Composite endpoint**	**Hazard ratios**	**95% confidence interval**	***p*-value**
BARC 5 bleeding events			
PAD group	Reference		
Unadjusted PAD group	1.141	0.476–2.730	0.771
Adjusted PAD group	0.591	0.229–1.523	0.276
BARC 3 bleeding events			
PAD group	Reference		
Unadjusted PAD group	2.511	1.757–3.588	<0.001
Adjusted PAD group	1.672	1.121–2.493	0.012
(**b**)			
**Composite endpoint**	**Hazard ratios**	**95% confidence interval**	***p*-value**
MACE			
PAD group	Reference		
Unadjusted PAD group	3.361	2.604–4.340	<0.001
Adjusted PAD group	2.152	1.510–3.066	<0.001
**Component endpoints**	**Hazard ratios**	**95% confidence interval**	***p*-value**
All-cause death			
PAD group	Reference		
Unadjusted PAD group	3.398	2.312–4.995	<0.001
Adjusted PAD group	1.768	1.149–2.722	0.010
Non-fatal myocardial infarction			
PAD group	Reference		
Unadjusted high PAD group	2.595	1.653–4.072	<0.001
Adjusted high PAD group	2.582	1.456–4.580	0.001
Readmission for heart failure			
PAD group	Reference		
Unadjusted PAD group	3.712	2.602–5.295	<0.001
Adjusted PAD group	2.118	1.261–3.560	<0.001

In the adjusted model, the PAD group (vs. Non-PAD group) was adjusted for age, gender, overweight (BMI ≥ 25), anemia, chronic renal failure on hemodialysis, previous myocardial infarction, previous cerebral infarction, CRP levels, STEMI, Killip class, diastolic blood pressure at admission, LVEF, use of NPPV, number of narrowed coronary arteries, first TIMI flow grade, use of drug eluting stents, and catheter size.

## Data Availability

The data that support the findings of this study are available from the corresponding author upon reasonable request.

## Supplementary Materials

The following supporting information can be downloaded at: https://www.mdpi.com/article/10.3390/jcm14093183/s1, Supplemental Table S1. Comparison of clinical characteristics between the matched PAD and matched non-PAD groups. Supplemental Table S2. Comparison of lesion and procedural characteristics between the matched PAD and matched non-PAD groups. Supplemental Table S3. Comparison of clinical outcomes between the matched PAD and matched non-PAD groups. Supplemental Figure S1. Kaplan-Meier curves for total bleeding or MACE-free survival events-free survival between the matched PAD and matched non-PAD groups.
