# Supplementary material for: The Association Between Peripheral Arterial Disease and Long-Term Bleeding Events in Patients with Acute Myocardial Infarction
Source: J Clin Med. 2025 May 4;14(9):3183. doi: 10.3390/jcm14093183 (PMC12072490; doi:10.3390/jcm14093183)
Supplement: Supplementary file 1 [file jcm-14-03183-s001.zip › jcm-3574889-supplementary.pdf]

Supplemental Table 1. Comparison of clinical characteristics between the matched PAD and matched non-PAD groups

	All (n=404)	Matched PAD (n=202)	Marched non-PAD (n=202)	*P* value
Age, years	77.0 (69.0-82.0)	77.0 (69.8-82.0)	76.0 (68.0-82.0)	0.288
Male, n (%)	295 (73.0)	149 (73.8)	146 (72.3)	0.823
Body mass index, (kg/m^2)	23.1 (21.0-25.4)	23.0 (20.6-25.5)	23.2 (21.2-25.3)	0.457
Ankle brachial index	0.99 (0.78-1.1)	0.78 (0.64-0.90)	1.10 (1.03-1.18)	<0.001
Brachial-ankle pulse wave velocity, (cm/sec)	1805 (1480-2193) (n=398)	1714 (1792-2233) (n=198)	1828 (1524-2132) (n=200)	0.794
Inter-arm blood pressure difference, (mmHg)	3.0 (1.0-7.0) (n=382)	4.0 (2.0-10.0) (n=191)	2.0 (1.0-4.0) (n=191)	<0.001
Current smoker, n (%)	115 (28.5) (n=403)	66 (32.8) (n=201)	49 (24.3)	0.061
Comorbidities				
Hypertension, n (%)	346 (85.6)	177 (87.6)	169 (83.7)	0.321
Hyperlipidemia, n (%)	252 (62.4)	127 (62.9)	125 (61.9)	0.918
Diabetes mellitus, n (%)	209 (51.7)	103 (51.0)	106 (52.5)	0.842
Anemia, n (%)	192 (47.5)	96 (47.5)	96 (47.5)	1.000
Atrial fibrillation, n (%)	76 (18.8)	36 (17.8)	40 (19.8)	0.703
Chronic renal failure on hemodialysis, n (%)	32 (7.9)	13 (6.4)	19 (9.4)	0.357
History of previous PCI, n (%)	108 (26.7)	55 (27.2)	53 (26.2)	0.911
History of previous CABG, n (%)	20 (5.0)	8 (4.0)	12 (5.9)	0.492
History of previous EVT, n (%)	42 (10.6)	42 (21.3)	0 (0.0)	<0.001
History of previous myocardial infarction,	73 (18.1)	36 (17.8)	37 (18.3)	1.000

n (%)				
History of cerebral infarction, n (%)	63 (15.6)	32 (15.8)	31 (15.3)	1.000
History of PAD surgery, n (%)	22 (5.4)	22 (10.9)	0 (0.0)	<0.001
Laboratory data				
Serum creatinine, (mg/dL)	0.96 (0.76-1.35)	1.03 (0.79-1.43)	0.91 (0.75-1.25)	0.090
eGFR, (mL/min/1.73 m2)	54.3 (35.8-70.6)	50.8 (34.4-67.1)	56.4 (40.1-74.3)	0.058
Hemoglobin levels, (g/dL)	12.8 (11.1-14.2)	12.8 (11.3-14.0)	12.8 (11.1-14.5)	0.688
Brain natriuretic peptide, (pg/mL)	306.0 (80.4-848.3) (n=400)	474.8 (101.0-927.0)	232.6 (64.4-752.7) (n=198)	0.008
Peak creatine kinase, (U/L)	437.0 (166.0-1289.3)	393.5 (149.0-1269.8)	480.5 (186.8-1337.3)	0.313
Peak creatine kinase-MB, (U/L)	31.0 (9.0-134.0)	26.0 (8.0-132.8)	34.5 (9.0-136.0)	0.242
Hemoglobin A1c, (%)	6.3 (5.8-7.3) (n=400)	6.3 (5.8-7.2)	6.3 (5.8-7.3) (n=198)	0.789
Platelets, ($\times 10^3/\mu L$)	21.5 (17.7-26.6)	22.4 (18.2-28.5)	21.2 (17.1-25.6)	0.050
C-reactive protein, (mg/μL)	0.30 (0.11-1.40)	0.46 (0.17-1.95)	0.19 (0.09-0.84)	<0.001
Type of acute myocardial infarction				
STEMI, n (%)	169 (41.8)	82 (40.6.)	87 (43.1)	0.687
NSTEMI, n (%)	235 (58.2)	120 (59.4)	115 (56.9)	
Cardiopulmonary arrest out of hospital, n (%)	12 (3.0)	5 (2.5)	7 (3.5)	0.771
Killip classification of 1 or 2, n (%)	284 (70.3)	143 (70.8)	141 (69.8)	0.913
Killip classification of 3 or 4, n (%)	120 (29.7)	59 (29.2)	61 (30.2)	
Cardiogenic shock at admission, n (%)	38 (9.4)	22 (10.9)	16 (7.9)	0.394
Vital sings				
Systolic blood pressure at admission,	141.0 (122.0-161.8)	140.5 (118.8-163.0)	141.0 (124.8-161.0)	0.621

(mmHg)				
Diastolic blood pressure at admission, (mmHg)	80.0 (69.0-94.0)	79.0 (66.6-92.0)	82.0 (70.0-97.0)	0.081
Heart rate at admission, (bpm)	84.0 (70.0-102.0)	85.0 (66.0-102.0)	83.0 (71.0-102.0)	0.779
Left ventricular ejection fraction, (%)	50.0 (36.3-61.3)	48.7 (35.8-61.2)	50.4 (37.0-61.0)	0.775
Medication at admission				
Aspirin, n (%)	148 (20.1) (n=399)	80 (40.2) (n=199)	68 (34.0) (n=200)	0.215
Thienopyridine, n (%)	98 (24.6) (n=399)	59 (29.6) (n=199)	39 (19.5) (n=200)	0.020
Statin, n (%)	175 (43.9) (n=399)	92 (46.2) (n=199)	83 (41.5) (n=200)	0.365
ACE inhibitors or ARBs, n (%)	201 (50.4) (n=399)	109 (54.8) (n=199)	92 (46.0) (n=200)	0.089
Beta-blockers, n (%)	131 (32.8) (n=399)	67 (33.7) (n=199)	64 (32.0) (n=200)	0.750
Calcium channel blocker, n (%)	181 (45.4) (n=399)	94 (47.2) (n=199)	87 (43.5) (n=200)	0.482
Diuretics, n (%)	109 (27.3) (n=399)	58 (29.1) (n=199)	51 (25.5) (n=200)	0.433
Oral antidiabetic, n (%)	140 (35.1) (n=399)	71 (35.7) (n=199)	69 (34.5) (n=200)	0.834
Insulin, n (%)	35 (8.8) (n=399)	19 (9.5) (n=199)	16 (8.0) (n=200)	0.601
Direct oral anticoagulants, n (%)	17 (4.3) (n=399)	10 (5.0) (n=199)	7 (3.5) (n=200)	0.470
Warfarin, n (%)	12 (3.0) (n=399)	5 (2.5) (n=199)	7 (3.5) (n=200)	0.771
Mechanical complications after PCI				
Ventricular septal perforation, n (%)	0 (0.0)	0 (0.0)	0 (0.0)	-
Cardiac free wall rupture, n (%)	0 (0.0)	0 (0.0)	0 (0.0)	-
Papillary muscle rupture, n (%)	1 (0.2)	1 (0.5)	0 (0.0)	1.000
Mechanical circulatory support				
Percutaneous cardiopulmonary support, n (%)	5 (1.2)	2 (1.0)	3 (1.5)	1.000
Intra-aortic balloon pumping, n (%)	166 (41.1)	62 (30.7)	104 (51.5)	<0.001

IMPELLA, n (%)	2 (0.5)	0 (0.0)	2 (1.0)	0.499
Medical therapy during hospitalization				
Temporary pacing, n (%)	13 (3.2)	10 (5.0)	3 (1.5)	0.087
Mechanical ventilation, n (%)	34 (8.4)	18 (8.9)	16 (7.9)	0.858
NPPV, n (%)	57 (14.1)	32 (15.8)	25 (12.4)	0.391
Continuous hemofiltration, n (%)	8 (2.0)	5 (2.5)	3 (1.5)	0.724

Supplemental Table 2. Comparison of lesion and procedural characteristics between the matched PAD and matched non-PAD groups

	All (n=404)	Matched PAD (n=202)	Matched non- PAD (n=202)	*P* value
Number of narrowed coronary arteries				0.403
Single, n (%)	150 (37.1)	72 (35.6)	78 (38.6)	
Double, n (%)	146 (34.7)	70 (34.7)	76 (37.6)	
Triple, n (%)	108 (26.7)	60 (29.7)	48 (23.8)	
Infarct-related artery				0.265
Left main-left anterior descending artery, n (%)	206 (51.0)	98 (48.5)	108 (53.5)	
Right coronary artery, n (%)	128 (31.7)	73 (36.1)	55 (27.2)	
Left circumflex artery, n (%)	65 (16.1)	29 (14.4)	36 (17.8)	
Graft, n (%)	5 (1.2)	2 (1.0)	3 (1.5)	
50% ≥ stenosis at left main coronary trunk, n (%)	55 (13.6)	31 (15.3)	24 (11.9)	0.384
First TIMI flow (0,1,2,3)				0.038
0, n (%)	96 (23.8)	56 (27.7)	40 (19.8)	
1, n (%)	28 (6.9)	9 (4.5)	19 (9.4)	
2, n (%)	87 (21.5)	37 (18.3)	50 (24.8)	
3, n (%)	193 (47.8)	100 (49.5)	93 (46.0)	
Final TIMI flow (0,1,2,3)				0.929
0, n (%)	2 (0.5)	1 (0.5)	1 (0.5)	
1, n (%)	3 (0.7)	2 (1.0)	1 (0.5)	
2, n (%)	9 (2.2)	5 (2.5)	4 (2.0)	
3, n (%)	390 (96.5)	194 (96.0)	196 (97.0)	
Chronic total occlusion in non-culprit arteries, n (%)	76 (18.8)	41 (20.3)	35 (17.3)	0.525

Use of aspiration catheter, n (%)	40 (9.9)	14 (6.9)	26 (12.9)	0.066
Final PCI procedure				0.743
Plain old balloon angioplasty, n (%)	13 (3.2)	7 (3.5)	6 (3.0)	
Drug-coated balloon, n (%)	40 (9.9)	24 (11.9)	16 (7.9)	
Bare metal stent, n (%)	5 (1.2)	3 (1.5)	2 (1.0)	
Drug eluting stent, n (%)	340 (84.2)	165 (81.7)	175 (86.6)	
POBA and thrombectomy, n (%)	1 (0.2)	0 (0.0)	1 (0.5)	
Aspiration only, n (%)	3 (0.7)	2 (1.0)	1 (0.5)	
Wire did not cross the lesion, n (%)	2 (0.5)	1 (0.5)	1 (0.5)	
Approach site				0.010
Radial, n (%)	247 (61.1)	126 (62.4)	121 (59.9)	
Brachial, n (%)	8 (2.0)	8 (4.0)	0 (0.0)	
Femoral, n (%)	149 (36.9)	68 (33.7)	81 (40.1)	
Catheter size (Fr)				0.120
6Fr, n (%)	258 (63.9)	121 (59.9)	137 (67.8)	
7Fr, n (%)	138 (34.2)	77 (38.1)	61 (30.2)	
8Fr, n (%)	8 (1.0)	4 (2.0)	4 (2.0)	

Supplemental Table 3. Comparison of clinical outcomes between the matched PAD and matched non-PAD groups

	All (n=404)	Matched PAD (n=202)	Matched non-PAD (n=202)	*P* value
Total bleeding event, n (%)	84 (20.8)	48 (23.8)	36 (17.8)	0.117
BARC type 3 bleeding, n (%)	66 (16.3)	39 (19.3)	27 (13.4)	0.138
-BARC type 3a bleeding, n (%)	52 (12.9)	27 (13.4)	25 (12.4)	0.882
-BARC type 3b bleeding, n (%)	25 (6.2)	17 (8.4)	8 (4.0)	0.097
-BARC type 3c bleeding, n (%)	1 (0.2)	0 (0.0)	1 (0.5)	1.000
BARC type 5 bleeding, n (%)	15 (3.7)	6 (3.0)	9 (4.5)	0.600
-BARC type 5a bleeding, n (%)	8 (2.0)	3 (1.5)	5 (2.5)	0.724
-BARC type 5b bleeding, n (%)	7 (1.7)	3 (1.5)	4 (2.0)	1.000
Bleeding site				
Gastrointestinal bleeding, n (%)	22 (5.4)	8 (4.0)	14 (6.9)	0.273

Intra-abdominal bleeding, n (%)	8 (2.0)	3 (1.5)	5 (2.5)	0.724
Access site-related bleeding, n (%)	14 (3.5)	11 (5.4)	3 (1.5)	0.053
Intracranial bleeding, n (%)	1 (0.2)	1 (0.5)	0 (0.0)	1.000
Required VA-ECMO, n (%)	5 (1.2)	3 (1.5)	2 (1.0)	1.000
Hematuria, n (%)	4 (1.0)	3 (1.5)	1 (0.5)	0.623
Others, n (%)	32 (7.9)	18 (8.9)	14 (6.9)	0.581
MACE, n (%)	121 (30.0)	80 (39.6)	41 (20.3)	<0.001
All-cause death, n (%)	58 (14.4)	36 (17.8)	22 (10.9)	0.064
-Cardiac death, n (%)	29 (7.2)	19 (9.4)	10 (5.0)	0.122
No-fatal myocardial infarction, n (%)	39 (9.7)	26 (12.9)	13 (6.4)	0.042
Re-admission for heart failure, n (%)	62 (15.3)	42 (20.8)	20 (9.9)	0.003

Supplemental Figure 1. Kaplan-Meier curves for total bleeding or MACE-free survival events-free survival between the matched PAD and matched non-PAD groups

A

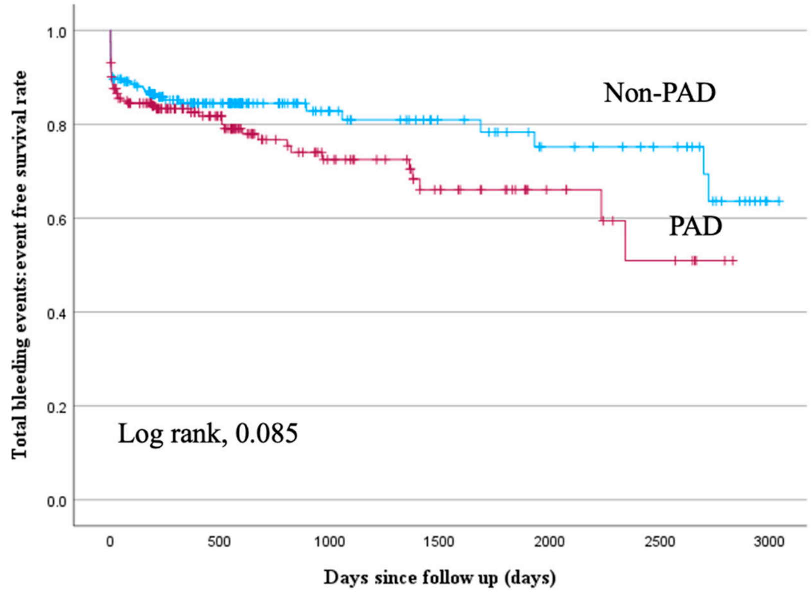

No.at risk

Non-PAD	202	95	45	27	12	6	0
PAD	202	99	45	32	22	17	1

B

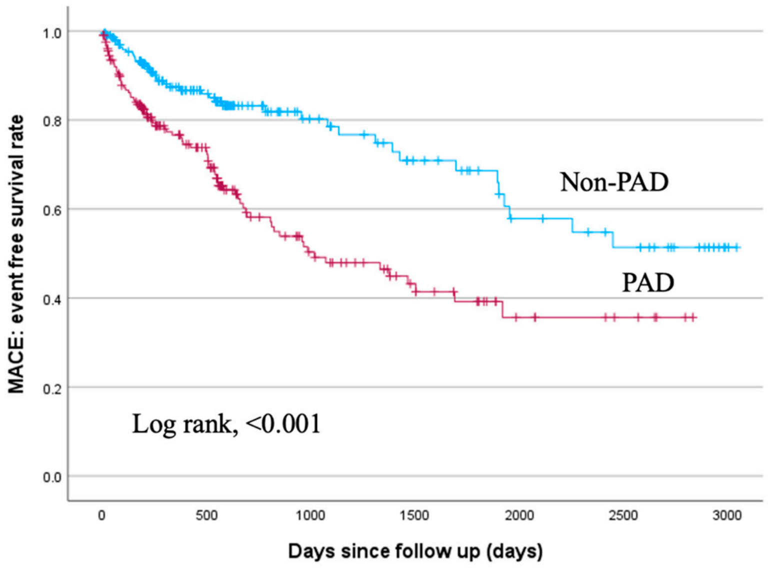

No.at risk

Non-PAD	202	104	48	27	20	15	2
PAD	202	97	42	24	22	9	0
